# Supplementary material for: Friends or foes? How activists and non-activists perceive and evaluate each other
Source: PLoS One. 2020 Apr 7;15(4):e0230918. doi: 10.1371/journal.pone.0230918 (PMC7138314; doi:10.1371/journal.pone.0230918)
Supplement: S1 Appendix — (DOCX) [file pone.0230918.s001.docx]

**S1 Appendix: Activists’ and non-activists’ perceptions of the issues in Study1 and Study 2**

We included a number of pre-measures to examine students’ perceptions of the issue and identification with relevant groups. More concretely, both activists and non-activists were asked to indicate to what extent they perceived the government measures to be unfair and immoral, identified with the broader group of students, but also specifically with those who opposed government measures (i.e. politicized, activist subgroup). Non-activists were also asked to indicate to what extent they identified with the group that supported government measures and whether they cared if the governmental measure were to be implemented. We also asked them whether they believed in the group’s efficacy to achieve its goals and felt personally affected by the government measures. All the items in the survey were measured on 7-point Likert-type scales ranging from 1- *Strongly disagree* to 7- *Strongly agree*.

Those who went to the demonstration perceived the government measures as highly unfair (*M* = 5.77, *SD* = 1.19) and immoral (*M* = 5.66, *SD* = 1.45). They identified highly with both the larger ingroup (*M* = 5.74, *SD* = 1.36) and with the politicized group (*M* = 5.72, *SD* = 1.38). Participants’ efficacy expectations were somewhat lower (*M* = 4.59, *SD* = 1.66). However, they indicated high motivations to attend future protests (*M* = 5.47, *SD* = 1.44). The activists perceived themselves to be relatively affected by these measures (*M* = 4.67, *SD* = 2.33).

In contrast, the sample of non-activists in Study 2 believed that the government plans were unfair (*M* = 4.64, *SD* = 1.50), and they cared about whether they would be implemented (*M* = 5.27, *SD* = 1.35). However, they did not see themselves as highly affected by these measures (*M* = 4.08, *SD* = 1.83), nor did they moralize the issue (*M* = 4.06, *SD* = 1.58). They identified highly with the broader ingroup, i.e., students as whole (*M* = 5.51, *SD* = 1.23). Their identification with the group which opposed (i.e., activist group *M* = 3.99, *SD* = 1.51) or supported the government (*M* = 3.13, *SD* = 1.32) were much lower. Most importantly, they had relatively little faith in students’ capability to influence the situation (*M* = 3.06, *SD* = 1.48) and they were not really motivated to join future protests (*M* = 2.37, *SD* = 1.31). This suggests that the non-activists did not have a strong stance on the issue and were neither strongly against nor in favour of the proposed government measures.
