# Supplementary material for: Friends or foes? How activists and non-activists perceive and evaluate each other
Source: PLoS One. 2020 Apr 7;15(4):e0230918. doi: 10.1371/journal.pone.0230918 (PMC7138314; doi:10.1371/journal.pone.0230918)
Supplement: S2 Appendix — (DOCX) [file pone.0230918.s002.docx]

**S2 Appendix: Activists’ perceptions of the COP21 protest in Study 3**

The participants of COP21 protest were asked to indicate to what extent their view of climate change is part of their moral values and norms, how highly they identify with those protesting against the negotiations (e.g. I identify highly/feel connected to/have strong ties with people who mobilized for COP protests, Cronbach’s α = .79), how angry they were at those who took part in the negotiations and to what extent they believed those who gathered influenced the negotiations. Similarly to the activist sample in Study 1, for people attending the COP 21 demonstrations climate change was a highly moralized issue (*M* = 5.91, *SD* = 0.88) and they were rather angry at the political powers taking part in these negotiations (*M* = 5.21, *SD* = 1.60). They identified highly with all the people who gathered there to protest the negotiations (*M* = 5.98, *SD* = 0.87), and they believed that these protests influenced the course of the negotiations (*M* = 4.99, *SD* = 1.38).
