# Supplementary material for: Friends or foes? How activists and non-activists perceive and evaluate each other
Source: PLoS One. 2020 Apr 7;15(4):e0230918. doi: 10.1371/journal.pone.0230918 (PMC7138314; doi:10.1371/journal.pone.0230918)
Supplement: S3 Appendix — (DOCX) [file pone.0230918.s003.docx]

**S3 Appendix: Activists’ and non-activists’ perceptions of the anti-nuclear weapon protest in Study 4**

We compared the activists and the M-turk sample included in the analyses on a set of premeasures (see Table below). Not surprisingly, the activists sample highly moralized the issue and was highly identified with the movement. However, the average scores above the mean point of the scale suggested that the M-turk sample of non-activists also perceived that the issue was unjust and believed that nuclear weapons. However, they did not feel personally affected nor they had high motivation to act.
