# Supplementary material for: Friends or foes? How activists and non-activists perceive and evaluate each other
Source: PLoS One. 2020 Apr 7;15(4):e0230918. doi: 10.1371/journal.pone.0230918 (PMC7138314; doi:10.1371/journal.pone.0230918)
Supplement: S4 Appendix — (DOCX) [file pone.0230918.s004.docx]

**S4 Appendix: The analyses with full sample in Study 4**

We ran a mixed effects ANOVA with Target Group (Target Activists vs. Target Non-activists) and Dimension (Selfishness vs. Morality vs. Irrationality) as within-subject factors and Participation (Activists vs. Non-activists) as a between-subject factor. The analysis yielded a significant effect for Dimension *F*(2, 200) = 53.95, *p <.* 001, η_p_^2^ = .35, significant Target Group x Dimension interaction *F*(2, 200) = 31.83, *p <.* 001, η_p_^2^ = .24, and most importantly a significant Target Group x Dimension x Participation interaction *F*(2, 200) = 13.50, *p <.* 001, η_p_^2^ = .12. The main effect of Target *F*(1, 200) = 1.95, *p =* .165, η_p_^2^ = .02, the interaction Target Group x Participation *F*(1, 200) = 1.41, *p =* .239, η_p_^2^ = .014, and Dimension x Participation *F*(2, 200) = 0.87, *p =* .42, η_p_^2^ = .01, were not significant.

In line with our expectations, activists perceived larger differences between two groups than non-activists. Concretely, they evaluated their own group as more selfless (*M*_targetactivists_ *=* 2.03, *SE* =0.31 vs. *M*_targetnon-activists_ *=* 3.44, *SE* =0.33), *F*(1, 100) = 18.36, *p <.* 001, η_p_^2^ = .16, and more moral (*M*_targetactivists_ *=* 5.83, *SE* =0.28 vs. M_targetnon-activists_ *=* 3.58, *SE* =0.31, *F*(1, 100) = 48.10, *p <.* 001, η_p_^2^ = .33. In contrast, non-activists perceived the two groups as equally selfless (*M*_targetactivists_ *=* 2.71, *SE* =0.16 vs. *M*_targetnon-activists_ *=* 2.93, *SE* =0.17), *F*(1, 100) = 1.69, *p =.* 197, η_p_^2^ = .02; and they thought the activists were more moral than non-activists: *M*_targetactivists_ *=* 4.67, *SE* =0.14 vs. *M*_targetnon-activists_ *=* 3.94, *SE* =0.16, *F*(1, 100) = 19.44, *p <.* 001, η_p_^2^ = .16. There were no differences in perceptions of irrationality. Means and standard deviations are reported in Table S2.

Mixed effects ANOVA yielded a significant effect for Dimension *F*(1,98) = 46.22, *p <.* 001, η_p_^2^ = .32, a significant Dimension x Participation interaction *F*(1,98) = 4.01, *p =.* 048, η_p_^2^ = .04, a significant Target Group x Dimension interaction *F*(1,98) = 56.38, *p < .*001, η_p_^2^ = .37, and a significant Target Group x Dimension x Participation interaction *F*(1,98) = 6.18, *p =.* 015, η_p_^2^ = .06. Main effect of Target *F*(1,98) = 2.70, *p = .*103, η_p_^2^ = .03, and interaction Target x Participation *F*(1,98) = 0.56, *p = .*457, η_p_^2^ = .006, were not significant.

Both activists and non-activists agreed that activists were more representative of those who care about the issue, whereas non-activists were representative of those who do not care about the issue. However, activists perceived larger differences between the two groups on both dimensions than non-activists
