# Supplementary material for: Friends or foes? How activists and non-activists perceive and evaluate each other
Source: PLoS One. 2020 Apr 7;15(4):e0230918. doi: 10.1371/journal.pone.0230918 (PMC7138314; doi:10.1371/journal.pone.0230918)
Supplement: S1 Table — (DOCX) [file pone.0230918.s005.docx]

**Table S1. Differences between activists and non-activists in Study 4**

|  | Participants: | | | | | | |
| --- | --- | --- | --- | --- | --- | --- | --- |
|  | Non-activists | | Activists | |  | | |
| *Evaluations of Targets*: | *M* | *SD* | *M* | *SD* | *t*- test | *df* | *p* |
| … believe that possession of nuclear weapons is unjust? | 4.72 | 1.85 | 6.42 | 0.84 | -5.58 | 67.36 | <.001 |
| ...see banning nuclear weapons as part of your moral values and beliefs ? | 4.49 | 1.93 | 5.89 | 1.63 | -2.87 | 78 | .005 |
| ...identify with those who believe that nuclear weapons should be banned? | 4.70 | 1.83 | 6.26 | 1.15 | -4.42 | 48.64 | <.001 |
| … identify with the movement Women’s march to Ban the Bomb? | 4.05 | 1.80 | 6.25 | 0.97 | -6.96 | 61.64 | <.001 |
| … intend to join future protests to ban nuclear weapons? | 3.30 | 1.61 | 6.05 | 1.32 | -6.94 | 79 | <.001 |
| …feel personally affected by the presence of nuclear weapons in USA? | 3.52 | 1.72 | 5.40 | 1.73 | -4.23 | 79 | <.001 |
