# Supplementary material for: Friends or foes? How activists and non-activists perceive and evaluate each other
Source: PLoS One. 2020 Apr 7;15(4):e0230918. doi: 10.1371/journal.pone.0230918 (PMC7138314; doi:10.1371/journal.pone.0230918)
Supplement: S2 Table — (DOCX) [file pone.0230918.s006.docx]

**Table S2. Study 4: Activists and general public views of both groups with full sample.**

|  | Participants: | | | |
| --- | --- | --- | --- | --- |
|  | Non-activists | | Activists | |
| *Evaluations of Targets*: | M | SD | M | SD |
| Selfish activists | 2.71 | 1.41 | 2.03 | 1.35 |
| Selfish non-activists | 2.93 | 1.49 | 3.44 | 1.57 |
| Moral activists | 4.67 | 1.37 | 5.83 | 0.90 |
| Moral non-activists | 3.94 | 1.50 | 3.58 | 1.14 |
| Irrational activists | 2.96 | 1.82 | 2.62 | 1.69 |
| Irrational non-activists | 2.56 | 1.60 | 3.38 | 1.56 |
| *Psychological distance:* |  |  |  |  |
| …activists representative of people who believe that nuclear weapons should be banned | 4.90 | 1.97 | 6.30 | 1.13 |
| …non- activists representative of people who believe that nuclear weapons should be banned | 3.54 | 1.65 | 3.50 | 1.79 |
| …activists representative of people who believe that nuclear weapons should not be banned | 2.69 | 1.79 | 2.05 | 1.99 |
| …non- activists representative of people who believe that nuclear weapons should not be banned | 3.79 | 1.70 | 4.15 | 1.42 |

*Note*. Unadjusted means (and standard deviations).

In sum, Study 4 showed that activists evaluated their own fellow group members as more selfless and moral than those who did not show up. The non-activists also appreciated those who went to protests, but in line with *Hypothesis 5* the differences in evaluations were consistently larger for the activist group suggesting that they tend to create more psychological distance from those who are not there, than vice versa.
