# Supplementary material for: Friends or foes? How activists and non-activists perceive and evaluate each other
Source: PLoS One. 2020 Apr 7;15(4):e0230918. doi: 10.1371/journal.pone.0230918 (PMC7138314; doi:10.1371/journal.pone.0230918)
Supplement: Attachment — Submitted filename: Response letter.docx [file pone.0230918.s008.docx]

Dear Dr. Wisneski,

Thank you again for giving us the opportunity to revise the manuscript. We hope we addressed your concerns regarding the theoretical distinctions between the hypotheses by providing clearer theoretical arguments for each one of them (see pages 6-8). Moreover, we felt our labeling of hypotheses as H1a & H1b for instance added to the confusion, because they could be interpreted as competing predictions, which is not the case here (therefore we relabeled them as Hypothesis 1 to 5). Moreover, the limitation section (page 35) now addresses another potential motivation for (in)action, namely emotions, which was not included in our research. Thus, we hope our manuscript is now better grounded in the social-psychological literature on collective action and will be of interest to the readership.

Thank you for your time and consideration. We look forward to hearing from you at the earliest time of your convenience.

Yours Sincerely,

Maja Kutlaca, Martijn van Zomeren & Kai Epstude
